# Admission NLPR predicts long-term mortality in patients with acute ischemic stroke: A retrospective analysis of the MIMIC-III database

**DOI:** 10.1371/journal.pone.0283356

**Published:** 2023-08-24

**Authors:** Xiao Su, Shigang Zhao, Nan Zhang

**Affiliations:** 1 Department of Neurology, Tianjin Medical University General Hospital, Tianjin, China; 2 Department of Neurology, Affiliated Hospital of Inner Mongolia Medical University, Hohhot, China; UMMC: The University of Mississippi Medical Center, UNITED STATES

## Abstract

**Background:**

The neutrophil to lymphocyte*platelet ratio (NLPR) is a new index based on platelets, neutrophils, and lymphocytes associated with the prognosis of patients with infectious diseases and cancer. However, its use in acute ischemic stroke has rarely been reported. This study examined the relationship between levels of systemic immunoinflammatory indices at admission and patient outcomes at different times after onset to assess stroke prognosis by NLPR.

**Methods:**

This was a retrospective cohort study. The data from 1222 stroke patients were obtained from multi-parameter intelligent monitoring in the Intensive Care III database(MIMIC- III). Cox proportional risk model was conducted to evaluate the relation between NLPR, all-cause mortality, and ischemic. The results were further verified via a subgroup analysis.

**Results:**

After adjusting for multiple covariates, it was found that NLPR was related with all-cause mortality in stroke patients. High NLPR was accompanied by an increase in mortality with longer follow-up (30 days: HR = 1.52, 95% CI = 1.14–2.02,90 days: HR = 1.67, 95% CI = 1.29–2.16, 365 days: HR = 1.56, 95% CI = 1.24–1.96 and 2 years: HR = 1.52, 95% CI = 1.22–1.89).

**Conclusion:**

The neutrophil to lymphocyte*platelet ratio (NLPR) are related to long-term adverse outcomes in patients with acute ischemic stroke. Therefore, NLPR is a promising inflammatory index for predicting the long-term prognosis of stroke.

## Introduction

Stroke is a major threat to human life and the second leading cause of death and disability worldwide [1, 2]. Ischemic strokes accounted for 87% of all strokes [3]. As the elderly population increases, the incidence of cerebral infarction is also on the rise resulting in higher rates of disability and mortality [4]. Every year 5.5 million people die of cerebral infarctionr [5]. Cerebral infarction has serious consequences such as death and disability if untreated promptly and appropriately. Therefore, it is critical to identify diagnostic and prognostic biomarkers for cerebral infarction.

The immune response influences the prognosis of acute ischemic stroke (AIS) [6]. Several studies have demonstrated that an inflammatory storm occurs in patients after stroke [7]. The brain’s inflammatory response after a stroke is a secondary mechanism of injury that can indirectly exacerbate brain damage [8]. The role of immune responses in ischemic stroke recovery has attracted the interest of clinical researchers [9]. Blood cells, including neutrophils, lymphocytes, monocytes, and platelets, are generally considered to reflect the inflammatory response of the body. Cell counts and their combinations, such as neutrophil-lymphocyte ratio (NLR), platelet-lymphocyte ratio (PLR), and derived neutrophil-lymphocyte ratio (dNLR), have recently emerged as classic hematological makers of systemic inflammation that can sensitively reflect the inflammatory response [10, 11]. These peripheral blood assays are readily available. NLR/PLR has been proven to have diagnostic value for various tumors, and has a good predictive effect on the prognosis of coronary atherosclerotic heart disease [12], sepsis, inflammatory bowel disease and other diseases [13–15]. A novel inflammatory biomarker, the neutrophil to lymphocyte*platelet ratio(NLPR), was constructed based on neutrophil, lymphocyte and platelet counts. NLPR can reflect the strength of the systemic immune response [16]. Since NLPR integrates data from neutrophil, platelet, and lymphocyte counts, examining these blood cells together can explore their interactions during AIS, although they may play opposing roles in AIS pathogenesis.

However, the underlying mechanism between inflammation and AIS remains unclear. Some results are inconsistent due to differences in study data. The association between NLPR and the long-term prognosis of ischemic stroke needs more clarification.

This study utilizes MIMIC-III database, which has good consistency and continuity of data to analyze the relationship between NLPR and the long-term prognosis of acute ischemic stroke.

## Materials and methods

### Data source

The data was obtained from the Intensive Care III database version 1.4 (MIMIC-IIIv1.4), a publicly available single-center critical care database. It has information on 46,520 patients admitted to the ICU at Beth Israel Deaconess Medical Center in Boston, Massachusetts, from 2001 to 2012 [17]. Information files comprise population chart events such as demographics, vital signs, lab tests, fluid balance, and vital status. Hospital staff recorded hourly physiological data from the International Classification of Diseases, Ninth Revision (ICD-9) code records verified by ICU nurses on bedside monitors at patient discharge. The database contains a written assessment of the corresponding period of radiology films by specialists. The documentation in the database was provided by clinicians, data scientists, information personnel, and users. The Institutional Review Boards of Beth Israel Deaconess Medical Center and the Massachusetts Institute of Technology (Cambridge University) approved this project. Patient information in this database was anonymized to protect the privacy of the patients. Therefore, informed consent was waived. An online training course was required prior to accessing the database. Su Xiao passed the exam to protect human research participants and was given access to the database (certification number:45610476) for data extraction.

### Patient selection

Patients over 18 with cerebral infarction were chosen using the International Classification of Diseases (ICD)-9 code. Patients with a history of transient ischemic attack (TIA) without developing into cerebral infarction and patients with WBC neutrophil-lymphocyte platelet mononuclear cell deficiency within 24 h of admission were excluded. If the patient was admitted to ICU more than once, only the data from the first admission was collected.

### Data extraction

The data were retrieved from MIMIC-III database, using Structured Query Language (SQL) and PostgreSQL software (version 9.6). The data encompassed demographic parameters, vital signs, laboratory parameters, co-morbidity parameters, date of death, date of ICU admission, and other variables. Co-morbidities included hypertension, cardiovascular disease (CVD), dyslipidemia, and diabetes mellitus. Laboratory data containing activated partial thromboplastin time (APTT), Prothrombin time(PT), creatinine, international normalized ratio (INR), lactate, red blood cell distribution width (RDW), white blood cells (WBC), lymphocytes, neutrophil, platelet, mononuclear cell, red blood cells (RBC), HDL, LDL, TC, TG, urea, the concentration of sodium, potassium, magnesium, chloride, and phosphate was also extracted. Vital signs such as diastolic blood pressure (DBP), systolic blood pressure (SBP), and heart rate were obtained. We collected these data during the first 24 hours after admission to the ICU. The NLPR [neutrophil/(lymphocyte ∗ platelet)] was calculated based on data obtained during the first 24 hours of each patient’s ICU admission. The endpoints of this study were all-cause mortality at 30 days, 90 days, 365 days, and two years from the date of ICU admission.

### Statistical analysis

The mean ± standard deviation was taken for continuous variables if the data were normally distributed (SD). The median (one, three, and four quartiles) was derived if the data were not normally distributed. Kolmogorov Smirnov test estimated whether the continuous data were normally distributed. Categorical variables were expressed as numbers or percentages. The Chi-square or Fisher’s exact test, one-way ANOVA, and Kruskal-Wallis H test determined significant differences between groups. Cox proportional hazards regression model were analyzed potential associations between NLPR and all-cause mortality at 30 days, 90 days, 365 days, and two years expressed as risk ratios (HRs) with 95% confidence intervals (CIs). Then, based on clinical co-morbidities and laboratory parameters, further subgroup analyses were performed to validate the role of NLPR on two-year all-cause mortality.

Variables such as demographic characteristics, co-morbidities, and laboratory tests were included as confounders in multivariate Cox regression analysis. Two multivariate models were used for each endpoint based on the NLPR group, identifying the first subgroup as the reference. In model I, these covariates were adjusted: age, race, sex, hypertension, cardiovascular disease, dyslipidemia, and diabetes mellitus. In model II, the following were accustomed: Thrombolytic Agent, creatinine, lactate, urea, RDW, C-reactive protein(CRP), red blood cells, potassium, phosphate, chloride, magnesium, sodium, APTT, PT, INR, TC, TG, HDL, LDL, systolic blood pressure, diastolic blood pressure, and heart rate. Subgroup analysis was performed using a multivariate Cox regression model. All the analyses were conducted using Stata software (version 16.0, CRAN), and SPSS software (v22.0; IBM, Armonk, NY), and P < 0.05 was defined as statistically significant. OriginPro 2019b plotted the histogram of mortality, and R version 4.1.3 was used to plot the forest map.

## Results

### Subject characteristics

**Fig 1:**
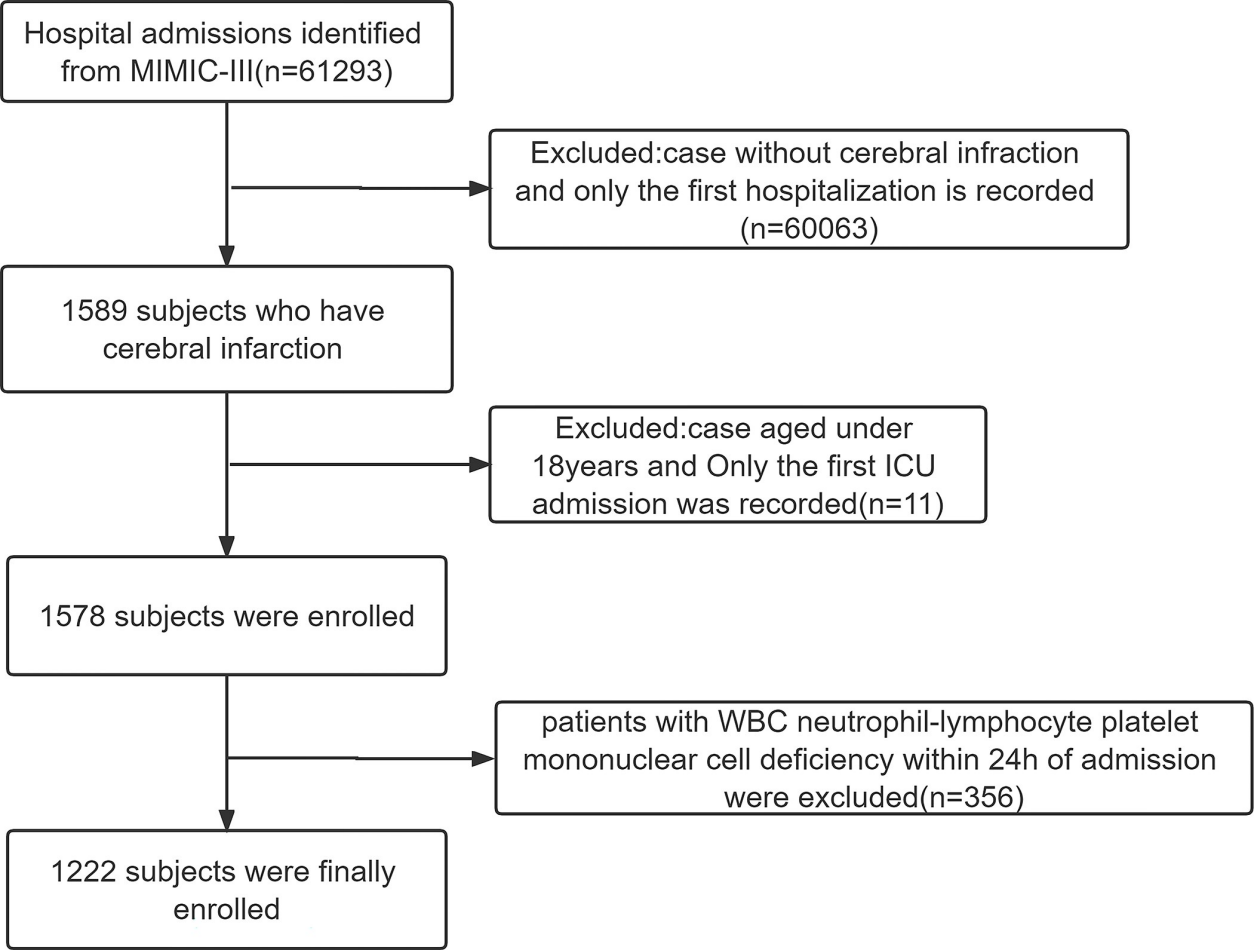
Flow diagram of selection for patients.

As per the selection criteria mentioned above, 1222 patients with cerebral infarction were included in this study (**Fig 1**). The patients were divided into three groups, and the basic information is listed in **Table 1**. Compared with the other two groups, patients with NLPR > 0.5 tended to have a history of Hypertension, hyperlipidemia, higher levels of systolic BP, heart rate, APTT, creatinine, INR, Lactate, PT, RBC, RDW, urea, CRP and Mg.

**Table 1:** Characteristics of the study patients according to NLPR.

Characteristics	NLPR			P-value
	≤0.2(n = 408)	0.3–0.4(n = 407)	≥0.5(n = 407)	
Age at onset, year, mean (SD)	67.27(16.60)	70.00(16.22)	69.82(15.54)	0.071
Gender,n(%)				
Female	239(58.6%)	206(50.6%)	187(45.9%)	0.001
Male	169(41.4%)	201(49.4%)	220(54.1%)	
Ethnicity,n(%)				
1 = WHITE	281(68.9)	299(73.5)	300(73.7)	0.086
2 = BLACK	51(12.5)	37(9.1)	28(6.9)	
3 = OTHER	76(18.6)	71(17.4)	79(19.4)	
Vital signs,mean (SD)				
Heart rate	80.75(15.83)	82.23(15.71)	85.79(16.73)	0.001
Systolic BP(mmHg)	133.43(19.89)	131.44(20.12)	128.34(19.96)	0.001
Diastolic BP(mmHg)	64.79(11.27)	64.35(12.42)	64.28(11.82)	0.801
Risk factors, n (%):				
Hypertension	258(36.4)	233(32.9)	217(30.6)	0.015
Diabetes	134(33.8)	131(33.0)	132(33.2)	0.980
Hyperlipidemia	173(37.9)	163(35.7)	120(26.3)	0.001
coronary atherosclerotic heart disease	97(33)	109(37.1)	88(29.9)	0.224
Thrombolytic Agent	35(8.6%)	39(9.6%)	47(11.5%)	0.353
Laboratory items mean (SD)				
APTT	26.10(23.80–29.50)	26.60(24.00–31.10)	27.90(24.00–33.40)	0.001
Creatinine	1.00(0.80–1.28)	1.00(0.80–1.40)	1.00(0.80–1.50)	0.002
TC, mmol/L	166.53 (45.46)	163.53 (46.81)	159.49 (45.59)	0.091
TG, mmol/L	151.32(184.18)	142.22(173.35)	151.87(185.81)	0.695
INR	1.10(1.00–1.30)	1.10(1.00–1.30)	1.20(1.10–1.40)	0.001
Lactate	2.18 (2.25)	1.97 (1.62)	2.47 (2.01)	0.001
PT	12.90(12.20–13.88)	13.20(12.50–14.70)	13.70(12.70–15.40)	0.006
RBC	4.21 (0.74)	4.21 (0.72)	4.12 (0.82)	0.154
RDW	14.31 (1.80)	14.37 (1.79)	14.68 (1.96)	0.008
Urea	19.00(14.00–26.00)	20.00(15.00–30.00)	21.00(15.00–34.00)	0.001
NA	139.10 (3.87)	139.36 (4.72)	139.11 (4.96)	0.578
K	4.27 (0.76)	4.27 (0.85)	4.21 (0.83)	0.466
Mg	2.00 (0.34)	1.99 (0.38)	1.91 (0.36)	0.002
HDL	45.28 (16.30)	42.67 (15.54)	43.90 (15.90)	0.065
LDL	92.68 (37.57)	89.16 (39.61)	91.99 (40.18)	0.397
Chloride	103.18 (4.64)	103.64 (5.78)	103.48 (6.32)	0.493
30-day mortality,n(%)	82/408(20.1%)	110/407(27.0%)	139/407(34.2%)	<0.001
90-day mortality,n(%)	97/408(23.8%)	135/407(33.2%)	179/407(44.0%)	<0.001
365-day mortality,n(%)	133/408(32.6%)	168/407(41.3%)	215/407(52.8%)	<0.001
2-Year mortality,n(%)	148/408(36.3%)	182/407(44.7%)	232/407(57.0%)	<0.001

### NLPR levels and all-cause mortality

**Fig 2:**
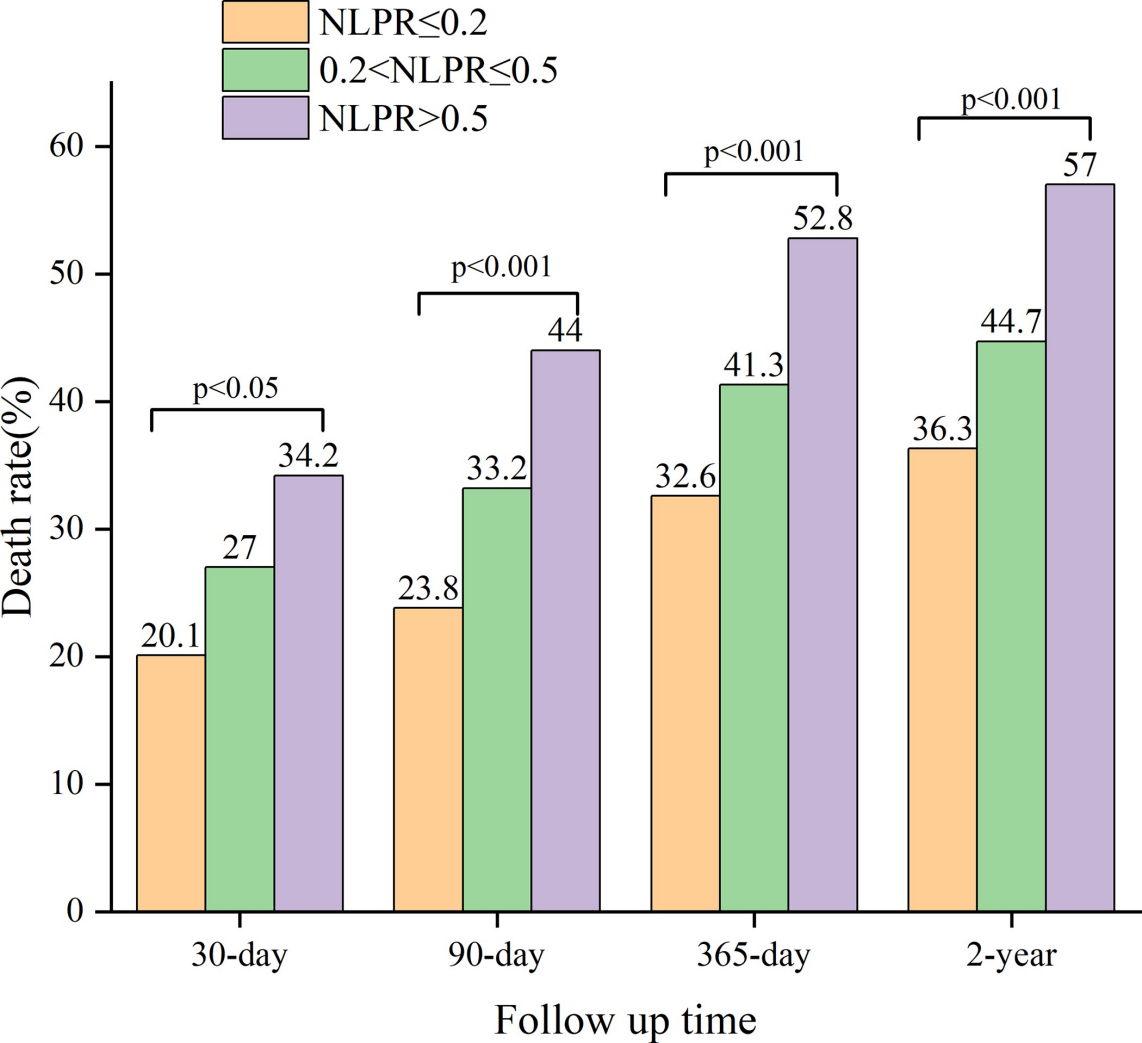
Relationship between NLPR and all-cause mortality in patients with cerebral infarction.

**Fig 3:**
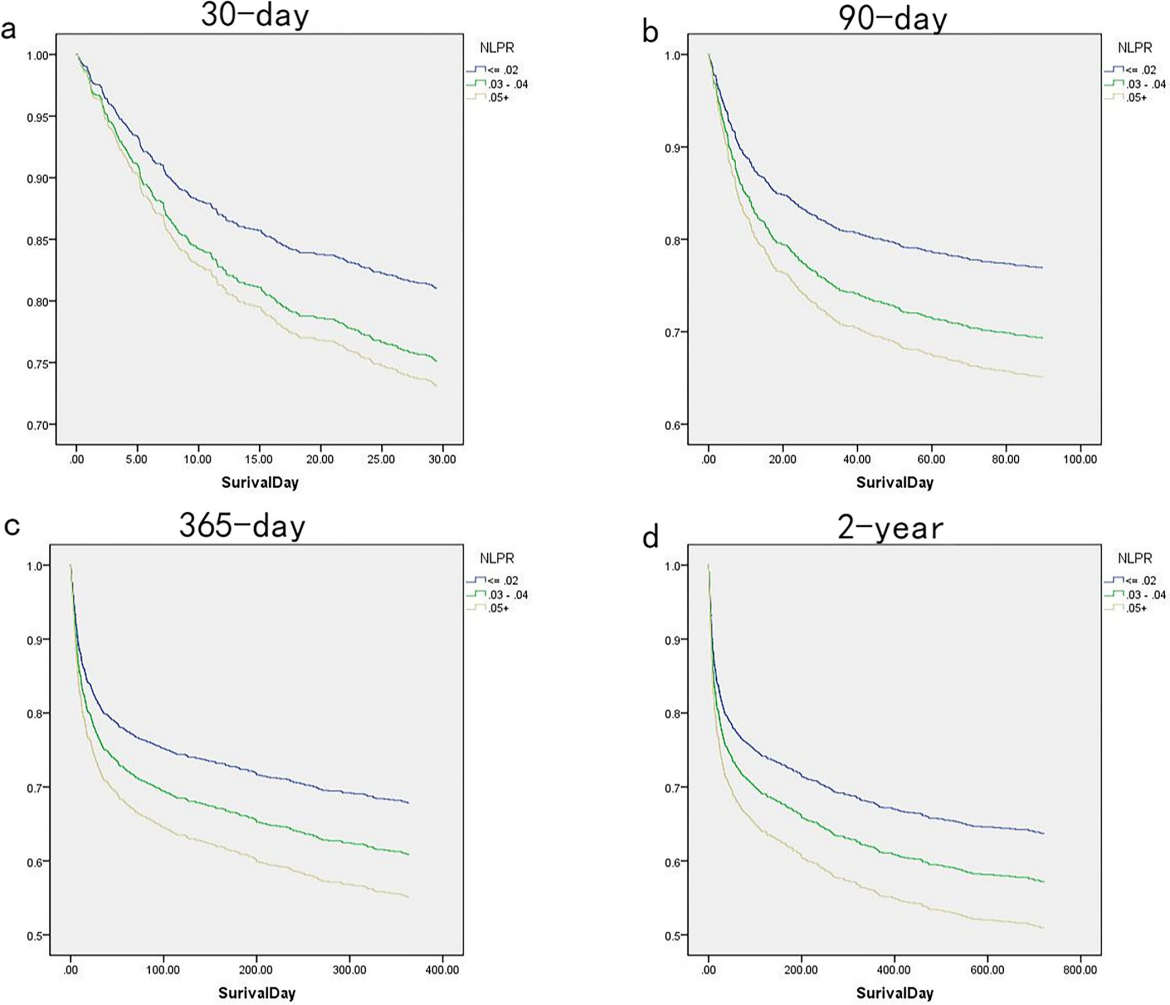
Probability of mortality curve for the patient with sepsis by NLPR. a. 30-day mortality; b. 90-day mortality; c. 1-year mortality; d. 2-year mortality.

**Fig 2** displays a significant difference in mortality from the low NLPR group to the high NLPR group for each endpoint. In a certain follow-up period, the mortality of patients with different NLPR levels was statistically different, hence, NLPR levels can be used as a biomarker for the prognosis of patients with acute severe cerebral infarction. The Cox proportional hazards regression models were used to determine different NLPR levels and all-cause mortality at 30 days, 90 days, one year, and two years in patients with cerebral infarction (**Table 2, Fig 3**). The lower NLPR group (NLPR ≤0.2) was used as a reference; high NLPR was related with an increased risk of all-cause mortality at 30 days, 90 days, 365 days, and two years in the Non-adjusted model (P for trend < 0.05). In model I, there was link between NLPR and 30-day,90-day, 365-day, and two-year all-cause mortality after adjusting for age, race, sex, hypertension, cardiovascular disease, dyslipidemia, diabetes, (P for trend < 0.05). After further adjustment for Thrombolytic Agent creatinine, lactate, urea, RDW, red blood cells, potassium, phosphate, chloride, magnesium, sodium, APTT, Creatinine, INR, TC, TG, Lactate, PT, RBC, RDW, NA, K, Mg, HDL, LDL, CRP, Chloride, systolic blood pressure, diastolic blood pressure, and heart rate in model II. There was a relationship between NLPR and all-cause mortality, and high NLPR with significantly associated with mortality with longer follow-up(30 days: HR = 1.52, 95% CI = 1.14–2.02, 90 days: HR = 1.67, 95% CI = 1.29–2.16, 365 days: HR = 1.56, 95% CI = 1.24–1.96 and 2 years: HR = 1.52, 95% CI = 1.22–1.89).

**Table 2:** HRs (95% CIs) for all-cause mortality across groups of NLPR.

	Non-adjusted		Model I		Model II	
Variable	HR (95% CIs)	*P*	HR (95% CIs)	*P*	HR (95% CIs)	*P*
		value		value		value
30-day all-cause mortality						
NLPR						
≤0.2	1.0 (ref)		1.0 (ref)		1.0 (ref)	
0.3–0.4	1.42(1.06,1.89)	0.017	1.34 (1.00,1.78)	0.048	1.38(1.03,1.84)	0.031
≥0.5	1.31(1.01,1.68)	0.001	1.64 (1.24,2.17)	0.001	1.52(1.14,2.02)	0.005
*P* trend		0.001		0.001		0.005
90-day all-cause mortality						
NLPR						
≤0.2	1.0 (ref)		1.0 (ref)		1.0 (ref)	
0.3–0.4	1.49 (1.15,1.93)	0.003	1.38(1.06,1.79)	0.017	1.42(1.09,1.85)	0.010
≥0.5	2.09 (1.64,2.68)	0.001	1.80(1.40,2.31)	0.001	1.67(1.29,2.16)	0.001
*P* trend		0.001		0.001		0.001
365-day all-cause mortality						
NLPR						
≤0.2	1.0 (ref)		1.0 (ref)		1.0 (ref)	
0.3–0.4	1.37(1.09,1.72)	0.007	1.26(1.00,1.58)	0.047	1.29(1.02,1.63)	0.031
≥0.5	1.92(1.55,2.38)	0.001	1.69(1.34,2.08)	0.001	1.56(1.24,1.96)	0.001
*P* trend		0.001		0.001		0.001
2-year all-cause mortality						
NLPR						
≤0.2	1.0 (ref)		1.0 (ref)		1.0 (ref)	
0.3–0.4	1.34(1.08,1.66)	0.008	1.23(1.00,1.53)	0.065	1.25(1.00,1.56)	0.050
≥0.5	1.89(1.54,2.33)	0.001	1.65(1.34,2.04)	0.001	1.52(1.22,1.89)	0.001
*P* trend		0.001		0.001		0.001

HR: hazard ratio; CI: confidence interval. Models were derived from Cox proportional hazards regression models. Non-adjusted model, adjusted for none. Adjust I model, adjusted for age, race, sex, hypertension, cardiovascular disease, dyslipidemia, and diabetes mellitus. Adjust II model, adjusted for age, ethnicity, gender, hypertension, cardiovascular disease, dyslipidemia, diabetes, creatinine, lactate, urea, RDW, red blood cells, potassium, phosphate, chloride, magnesium, sodium, APTT, Creatinine, INR, TC, TG, Lactate, PT, RBC, RDW, NA, K, Mg, HDL, LDL, CRP, Chloride, systolic blood pressure, diastolic blood pressure, and heart rate and thrombolytic agent infusion therapy.

### Subgroup analysis

**Fig 4:**
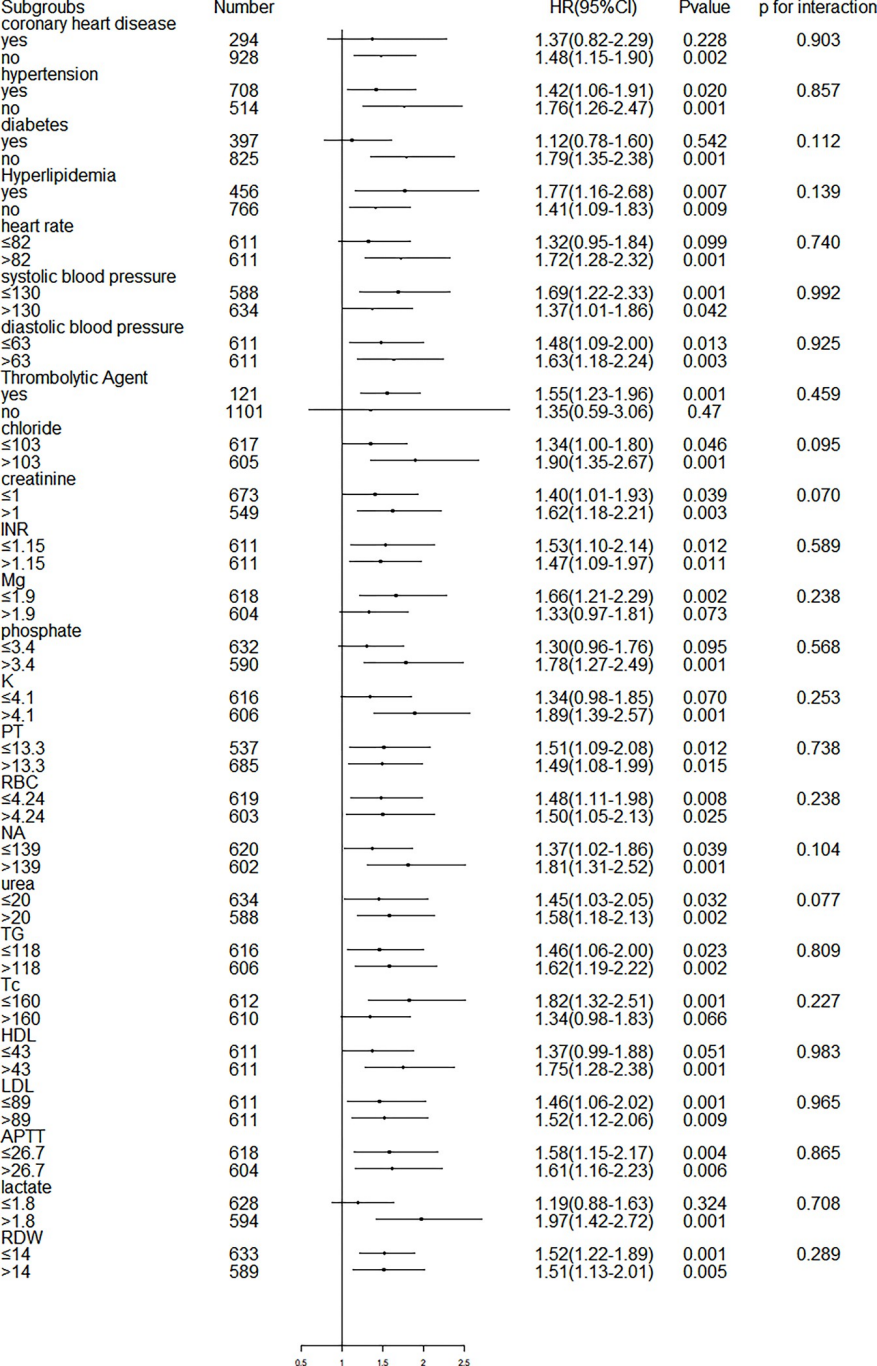
Subgroup analysis of associations between NLPR and 2-year all-cause mortality based on different comorbidities and laboratory parameters levels.

Subgroup analyses of laboratory parameters and co-morbidities were performed by level, as shown in **Fig 4**. NLPR levels were similar to two-year all-cause mortality in most strata, and there was no interaction (P > 0.05).

## Discussion

This was a retrospective study of an ICU inpatient population. This study investigated the relationship between NLPR and the prognosis of patients with acute ischemic cerebral infarction. The findings of the current study stated that NLPR has a good predictive value for the long-term prognosis of AIS patients, and high NLPR is strongly related to the poor long-term prognosis in stroke patients. Therefore, NLPR is suggested to be a new indicator for the prognosis of treated patients of AIS. A probable explanation for the relationship between NLPR and poor long-term prognosis in patients with acute ischemic cerebral infarction may be the immune changes during acute ischemic stroke. Considering that stroke is associated with inflammation [11]. NLPR, a comprehensive systemic marker of inflammation consisting of neutrophil, lymphocyte, and platelet counts, is suggested to be a new indicator of long-term prognosis in acute severe cerebral infarction patients.

Many studies have demonstrated the importance of inflammation in the pathogenesis of stroke [18]. The ischemic cascade response results in the release of factors such as neuronal cell death and damage-related molecular patterns, ensuing in a local inflammatory response in the damaged region of the brain [19]. Intravascular injury leads to vascular occlusion, and inflammatory cells converge into the brain parenchyma, causing tissue damage and blood-brain barrier impairment. When the blood-brain barrier is disrupted, peripheral blood leukocytes infiltrate into the damaged brain region, further disrupting the blood-brain barrier by periodically releasing pro-inflammatory cytokines, reactive oxygen species, and matrix metalloproteinases [20]. Studies have shown that neutrophils are the first innate immune cells to respond to cerebral ischemia [21]. Neutrophils infiltrate the ischemic site within hours after AIS and reach peak neutrophil concentrations after 24-48h [22], leading to elevated expression of matrix metalloproteinase-9, which further damages brain parenchyma [23]. In clinical studies, FERRO et al. showed in 553 patients with acute cerebral infarction that the ratio of neutrophils to lymphocytes increased with the degree of cerebral edema at 24 h and correlated with early neurological deterioration and functional status at 90 days [24]. Wei et al. found that neutrophils were increased in peripheral blood shortly after stroke onset in stroke patients and that higher neutrophil counts indicated a harmful stroke prognosis. Neutrophil elevation in peripheral blood peaked in stroke mice 1 day after stroke and then gradually decreased to the level of sham-operated mice by day 2 [25]. Moreover, neutrophils aggravate brain damage by releasing inflammatory mediators [26]. After AIS, neutrophil infiltration correlates with the severity of the injury. Following AIS, blood stagnation flow exerts shear stress on endothelial cells, resulting in the deployment of p-selectin, an adhesion molecule, to the cell surface [22]. Platelets interact with circulating leukocytes via modifying the platelet surface, producing platelet leukocyte aggregates and promoting leukocyte aggregation, which leads to intravascular occlusion and ischemic injury [27]. In addition, other immune cells also play important roles in ischemic stroke. For example, cerebral ischemia and hypoxia can stimulate monocytes to yield inflammatory mediators: interleukin-6 (IL-6) and tumor necrosis factor (TNF), exacerbating cerebral ischemia and hypoxia, resulting in more extensive cerebral tissue destruction [28]. Lymphocytes are involved in the inflammatory response following stroke. However, the mechanism of lymphocyte action in AIS is debatable.

Some animal stroke experiments have reported that lymphocytosis upregulates IL-10 levels and inhibits inflammatory cytokines such as IL-6 and TNF-α, thus exerting neuroprotective effects [29, 30]. Previous research has shown that lymphocytes exacerbate the inflammatory response and brain damage after stroke [31]. Furthermore, T cells and γδ TCD4+ and CD8+ cells contribute to AIS by producing pro-interferon and pro-inflammatory cytokines like IL-17 [32]. Moreover, natural killer cells exert neuroprotective effects by secreting IL-10 through regulatory T cells (Tregs) [33]. Stroke induces activation of immune and inflammatory pathways both in the central nervous system (CNS) and periphery, characterized by microglial activation, recruitment of leukocytes, and activation of the hypothalamic-pituitary-adrenal axis and autonomic nervous system. In parallel, a simultaneous suppression of systemic cellular immune function accompanies an activation of systemic inflammatory response [7, 34]. Frøyshov et al. showed that AIS patients with higher leukocytes, fibrinogen, IL-6, and hs-CRP at baseline were associated with higher all-cause mortality at 16 years of follow-up [35]. This suggests that elevated inflammatory markers predict long-term mortality in ischemic stroke survivors. Therefore, the inflammatory response to AIS is highly complex. Inflammatory responses aggravate ischemic brain injury and neurological dysfunction, while chemokines and cytokines released from ischemic tissue boost the infiltration of peripheral circulating leukocytes into ischemic sites [36]. Leukocyte infiltration and the release of various inflammatory mediators cause neuronal death or apoptosis, resulting in poor outcomes in AIS patients.

The above-stated mechanism elucidates why several inflammatory parameters based on their association with stroke are valuable in stroke prognosis, such as neutrophil-lymphocyte ratio (NLR) and platelet-lymphocyte ratio (PLR) [37]. Studies have revealed that NLR is positively correlated to the risk of death at three months in stroke patients [38]; increased PLR predicts the post-stroke depression [39]; low LMR is independently allied with the risk of hemorrhagic transformation in stroke patients [40], and higher RDW can independently forecast stroke patients ending [41]. A single inflammatory index is insufficient due to its simple structure to reflect the severity of inflammation. Inflammation closely accompany stroke. NLPR, which combines platelet count, neutrophils, and lymphocytes, as a marker of systemic immune inflammation could be a good predictor of long-term prognosis in AIS.

Finally, understanding the inflammatory response mechanism after stroke has important application value for immunomodulatory therapy. NLPR combines neutrophils, lymphocytes, and platelets to better reflect the systemic inflammatory response. NLPR is an independent predictor of adverse long-term outcomes in stroke patients. No previous study, to our knowledge, has explored the role of NLPR in the prognosis of acute severe ischemic stroke. Therefore, NLPR as a predictor of stroke prognosis has great potential.

### Limitations

This study has some limitations. First, the subjects of this study were from a single monocentric, which may have resulted in selection bias or geographic bias; the next step will be to undertake a multicenter study. Second, there are also some factors, such as sex, that may contribute to changes in NLRP. This may be because estrogen can increase the aggregation of neutrophils and monocytes, promote the formation of megakaryocyte polyploids, increase the number of lymphocytes [42, 43], and promote the formation and release of PLT precursors [44, 45]. However, this is consistent with our results that at the low NLPR level there are more men and fewer women, and at the high NLPR level there are fewer men and more women. Other influencing factors were studied in subgroup analyses. The results showed no significant interaction between the other factors. Furthermore, the number of covariates associated with stroke prognosis was very large and not collected adequately in this study. And future studies should document in detail the causes of death after stroke. More data on other parameters are required to improve the current study results.

## Supporting information

S1 Data (ZIP)
